# Single-Tube Reaction Using Perfluorocarbons: A Prerequisite Step Leading to the Whole-Slide In Situ Technique on Histopathological Slides

**DOI:** 10.1371/journal.pone.0158018

**Published:** 2016-06-23

**Authors:** Yi-Chang Chen, Tsung-Han Teng, Jane S.-C. Tsai, Hsien-Da Huang, Yih-Leong Chang, Cher-Wei Liang

**Affiliations:** 1 Biomedical Technology and Device Research Laboratories, Industrial Technology Research Institute, Hsinchu, Taiwan; 2 Institute of Molecular Medicine and Bioengineering, National Chiao Tung University, Hsinchu, Taiwan; 3 Department and Graduate Institute of Pathology, National Taiwan University Hospital and National Taiwan University College of Medicine, Taipei, Taiwan; University of Toronto, CANADA

## Abstract

Developing a robust, novel method for performing multiple reactions in a single tube is not only time- and cost-saving but also critical for future high-throughput whole-slide in situ techniques on diseased tissues. In this study, we introduce the use of perfluorocarbons and compound-coated magnetic particles to create pseudochambers in a single tube, allowing different reactions to be performed in different phases. Perfluorocarbons also serve as cell lysis buffer and polymerase chain reaction (PCR) buffer owing to their highly penetrating, repellent and emulsifiable properties. Using this method, nucleic acids can be isolated and purified from various sample types and sizes, followed by PCR, real-time PCR, or multiplex PCR in the same tube. No incubation or enzyme digesting time is needed and the risk of cross-contamination is reduced. Tests can be performed in microemulsions (water-in-oil droplets) containing sequence-specific captures and probes for further high-throughput detection. We present a simple, quick, and robust procedure as a prerequisite step to future high-throughput in situ techniques.

## Introduction

Histopathology slides harbor topological information on disease tissues. Like liquid-crystal display and e-ink paper [1], if a histopathology slide could be gridded into hundreds to thousands of non-communicating cells and work independently (as in Eppendorf tubes) for tissue lysis, nucleic acid/protein extraction, and liquid-phase-based multiplex reactions, a digital slide containing disease information could be created. The combination of the topological information provided by solid-phase disease tissue and the advantage of efficiently conducting multiplex reactions in liquid-phase state has great potential in detailing the spatial regulation and interaction of different molecules important in disease development and progression. Furthermore, if real-time or emulsion polymerase chain reaction (PCR) could be introduced into each cell, disease molecules of interest at certain slide coordinates could be quantified. [2, 3]

The first critical step in achieving this aim is to discover an efficient way to perform multiple reactions, including nucleic acid isolation, amplification, and detection, in a single tube. This will enable the simulation of immobilized cells on slides after gridding as changing thousands of in situ tubes will not be feasible. In this study, we introduce the use of perfluorocarbons (PFC) and magnetic particles to achieve this goal. Compared with the conventional methods, using only one tube greatly simplified the procedures and reduced the risk of cross-contamination, and the isolated and amplified nucleic acids were of comparable quality. No incubation or enzyme digesting time was needed. After isolation, reagents for use in nucleic acid amplification were added directly into the same tube without further treatment. Tests could be performed in microemulsions (water-in-oil droplets) containing sequence-specific captures and probes for further high-throughput detection. The overall procedure is simple, quick, and robust and is a prerequisite step leading to high-throughput in situ techniques in the future.

## Materials and Methods

### Single-tube reaction for RNA isolation

**Fig 1:**
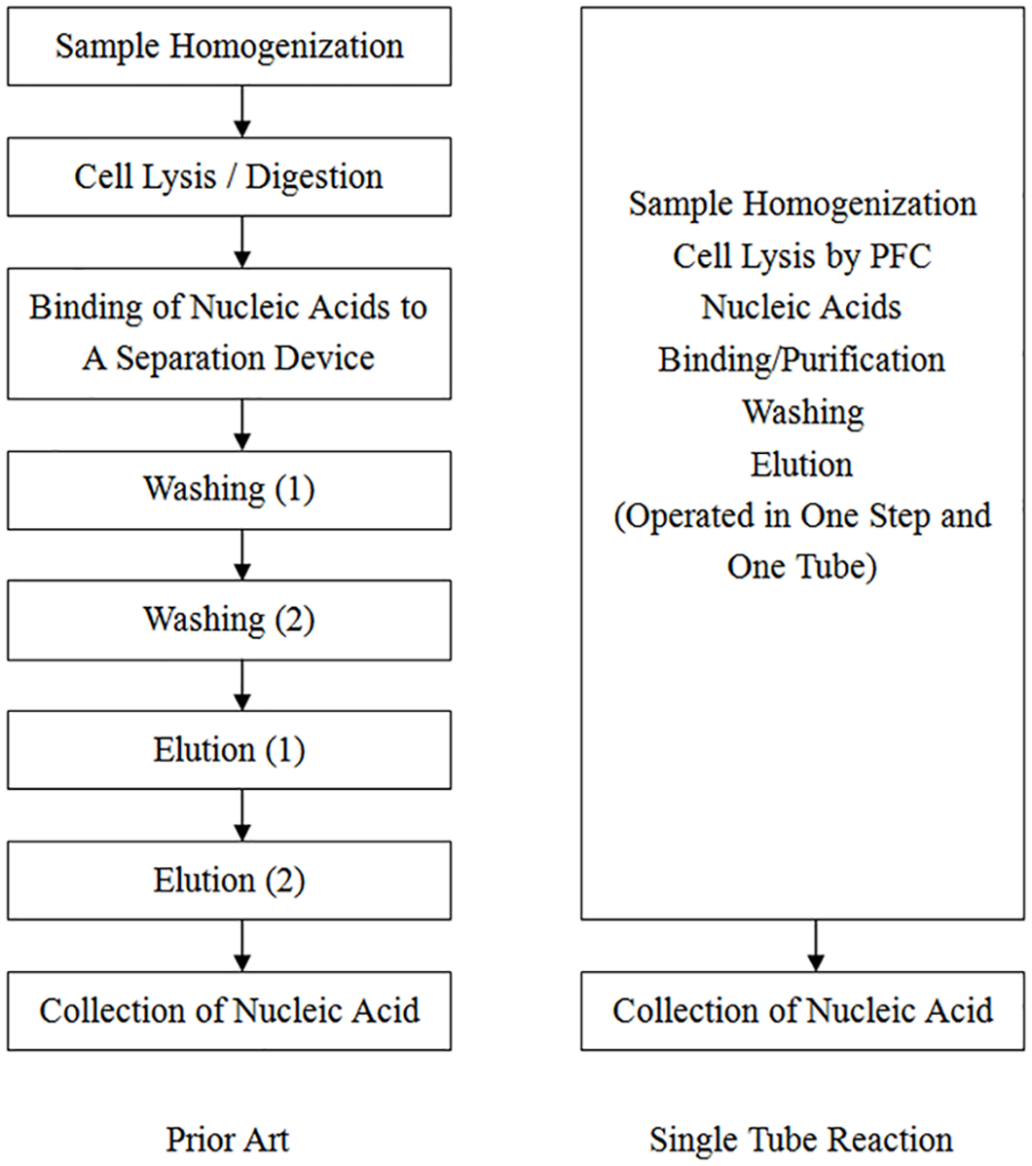
Overall flow chart comparing conventional and single-tube methods. We introduce the use of perfluorocarbon (PFC) for cell lysis and magnetic particles for nucleic acid purification in single-tube reaction. Compared with the conventional methods, using only one tube greatly simplify the procedures and reduce the risk of cross-contamination. No incubation or enzyme digesting time is needed.

The overall flow chart is shown in Fig 1, which compares conventional and current single-tube methods. 1 mg of ground or non-ground mouse liver tissue was tested for nucleic acid isolation. Lysis buffer [1:1 mixture of perfluorohexane (Fluorinert^™^) and a mix aqueous solution of 4.5 M LiCl and 5 M GuCl with 1% Triton X-100, maintained at a pH > 12] and wash buffer (3.5 M LiCl in 70% ethanol) were prepared. The mouse liver tissue was added into a centrifuge tube containing 1000 μl lysis buffer, briefly mixed, followed by the addition of 1000 μl wash buffer and 100 mg magnetic beads (homemade, 1 μm, dextran-coated, positively charged). Briefly centrifuge the tube (1000 rpm) to obtain an upper aqueous phase and a lower organic phase. The tube was then intensely vortexed for 5 min to achieve a homogenized state. The nucleic acid-binding magnetic beads in the mixed solution were then separated with magnetic stage (Ambion) and eluted with a gradient of diethyl pyrocarbonate aqueous solution from low pH (to remove alcohol) to high pH (to isolate nucleic acid from dextran-coated beads) to obtain a purified sample (named ITRI sample). The beads were removed, and products were compared with those from commercialized RNA isolation kits (Qiagen RNAeasy^®^, named Qiagen sample).

The same procedure was used to isolate RNA from varying amounts of mouse solid tissues (0.1–1 mg tissues from liver, heart, spleen, kidney, lung, and femur) and fluids (10 μl of mouse blood, sera, and urine). Lysis buffer was adjusted with the solid sample amount. For hard tissues, such as bone (femur) and cartilage (lung), an additional grind step was performed on the tissue in lysis buffer before adding wash buffer. For the fluids, lysis buffer was adjusted with 12.5 μl perfluorohexane (Fluorinert^™^), 10 μl polytetramethylene glycol (MW 8000), and 0.1 μl Triton X-100 to increase nucleic acid precipitation. For comparison, a technical duplicate that only differed in RNA-collecting (purification) method magnetic beads vs. precipitate centrifugation was performed. RNA integrity number (RIN) was checked by Agilent 2100^®^ platform. The effects of variation in perfluorohexane ratio of lysis buffer were checked.

### Single-tube nucleic acid amplification

10 μl of mouse blood and 10 μl mouse sera were prepared in lysis buffer as described above. The mixture was incubated for 3 min and then mixed with 12.5 μl amplification reagents (10 mM (NH_4_)_2_SO_4_, 10 mM KCl, 2 mM MgSO_4_, 20 mM of 0.1% Triton X-100, Tris-HCl pH 8.8, and polymerases). The selective gene *GAPDH* was amplified by adding 1 mM of commercially available primer pairs (Fermentas, standard GAPDH primers) into the same tube. PCR was performed using a PCR machine (ABI 9700) at the following PCR conditions: (1) 95°C for 15 min, (2) 95°C for 10 s, 58°C for 30 s, and 72°C for 30 s for 40 cycles, and (3) 72°C for 7 min. The amplified products were run on electrophoresis gel (Tris-acetate-EDTA gel, 75 V) and compared with those from using commercial kits (Fermentas Maxima^®^ reagent kit).

### Single-tube nucleic acid amplification using various sample volumes

The samples were prepared as above, but a volume of 1, 10, 15 and 20 μl mouse blood was used. The ratio of each component is listed in Table 1.

**Table 1:** Single-tube nucleic acid amplification using various sample volumes.

No.	Lysis buffer (μl)	Sample (μl)	Amplification reagent (μl)	Ratio of lysis buffer in the total volume of mixture (%)
1	1	1	48	2
2	10	10	30	20
3	15	15	20	30
4	20	20	10	40

### Single-tube real-time PCR amplification

Sample/lysis buffer mixtures were mixed with 1 mM 18S/GAPDH primer pairs (ABI PCR control, standard 18S/GAPDH primers) and submitted for real-time PCR. The PCR conditions were set as follows: denaturation at 95°C and annealing at 58°C in one cycle, repeated for 40 cycles. For further application tests, standard dengue virus sera samples were used by adding dengue standard primer pairs (FDA standard primers). The PCR conditions were set as follows: 95°C for 15 min; 95°C for 30 s, 60°C for 30 s, and 72°C for 30 s in one cycle, repeated for 40 cycles. Multiplex PCR amplification was then tested by simultaneously adding two sets of primers into the single-tube mixture: 1 mM of GAPDH and β-actin primer pairs (ABI PCR control Primer Beta-actin 435234E, GAPDH 4308313). The PCR conditions were set as follows: 95°C for 15 min; 95°C for 10 s, 60°C for 30 s, 70°C for 30 s, and 72°C for 30 s in one cycle, repeated for 35 cycles. Various solid plant tissues were also tested, including 1 mg of *Semen Coicis*, *Vigna umbellata*, *Adenanthera pavonina*, rice, and brown rice tissue samples. The sample/lysis buffer mixture was directly amplified with NCBI plant identification standard primers. The PCR conditions were set as follows: denature at 95°C and anneal at 60°C in one cycle, repeated for 40 cycles.

### Single-tube emulsion PCR amplification

To prepare oil-surfactant mixture, the oil-surfactant components (S1 Table) were thoroughly mixed in a 50 ml-centrifuge tube at 25°C. 400 μl of the oil-surfactant mixture was moved to Cryo Tube ampoules and 3 × 8 mm magnetic stir bars were added. The mixture was blended with the stir bars at 1,000 rpm. The water phase components (S2 Table) were then mixed to form the water phase of the emulsion, i.e., the PCR reaction buffer. To test single-tube emulsion PCR, 10 μl lysis buffer [1 μl perfluorohexane (Fluorinert^™^), 8 μl polytetramethylene glycol, and 1 μl Triton X-100] was added to 20 μl whole blood and mixed and incubated for 3 min. For solid tissue, an additional step of centrifuge or washing (with magnetic beads) was performed to remove debris. 20 μl of oil (oil-surfactant) and water phase PCR components (see above) were directly introduced into the same tube. An emulsion was formed in which PCR reagents and nucleic acids were encapsulated within oil droplets. A microscopic photograph (at 50-fold magnification) of the emulsion could be taken at this time to ensure the encapsulation of fluorescence-labeled nucleic acids. The lysis buffer and the PCR reaction buffer could be added separately or pre-mixed before addition. The emulsion was then amplified with emulsion PCR under the following conditions: 95°C for 15 min, 94°C for 45 s, 60°C for 45 s, 72°C for 60 s in one cycle, repeated for 40 cycles. The solution was then kept at 72°C for 5 min and stored at 4°C for optical analyses.

**Fig 2:**
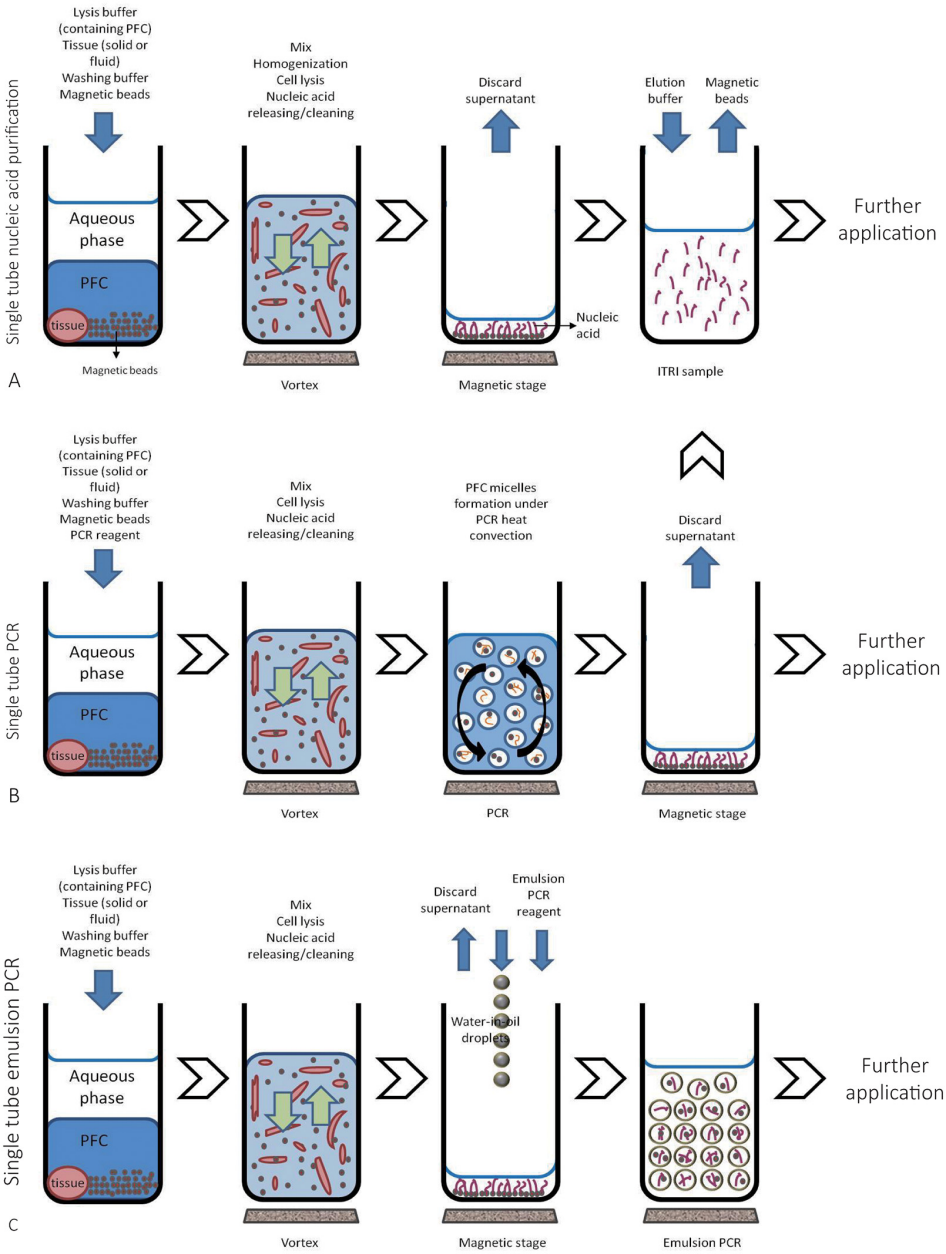
Summary of single-tube reactions. (A) Single-tube nucleic acid purification. All reagents (including lysis buffer containing PFC and wash buffer) can be added in one step, greatly simplifying the procedure. After a brief and intense vortex a homogenization state is achieved (see discussion). Nucleic acids are then purified by 1 μm, dextran-coated and positively charged magnetic beads. (B) Single-tube PCR. After isolation, reagents for use in nucleic acid amplification can be added directly into the same tube without further treatment, or, all the reagents required for nucleic acid isolation and amplification (including lysis buffer, washing buffer and PCR reagents) can be added in one step in the very beginning of the experiment. PCR is then conducted in PFC micelles. The amplified products are collected and purified by coated magnetic beads. (C) Single-tube emulsion PCR. After cell lysis and magnetic bead purification of nucleic acids, reagents for emulsion PCR can be added directly into the same tube after discarding the supernatant and the cellular debris. Tests can be performed in microemulsions (water-in-oil droplets) containing sequence-specific captures and probes for further high-throughput detection. The tube sizes in each column are not to scale.

A cartoon summary of the methods is shown in Fig 2. All the animal tissues used in this study were purchased via Industrial Technology Research Institute (Hsinchu, Taiwan) from BioLASCO Taiwan Co., Ltd (Taipei, Taiwan) with Full Accreditation of AAALAC International, under the regulations of ITRI Institutional Review Board (IRB). The project was approved by the IRB of the National Taiwan University Hospital (REC No.: 201412155RIND).

## Results

### Analyses of isolated RNA samples

**Fig 3:**
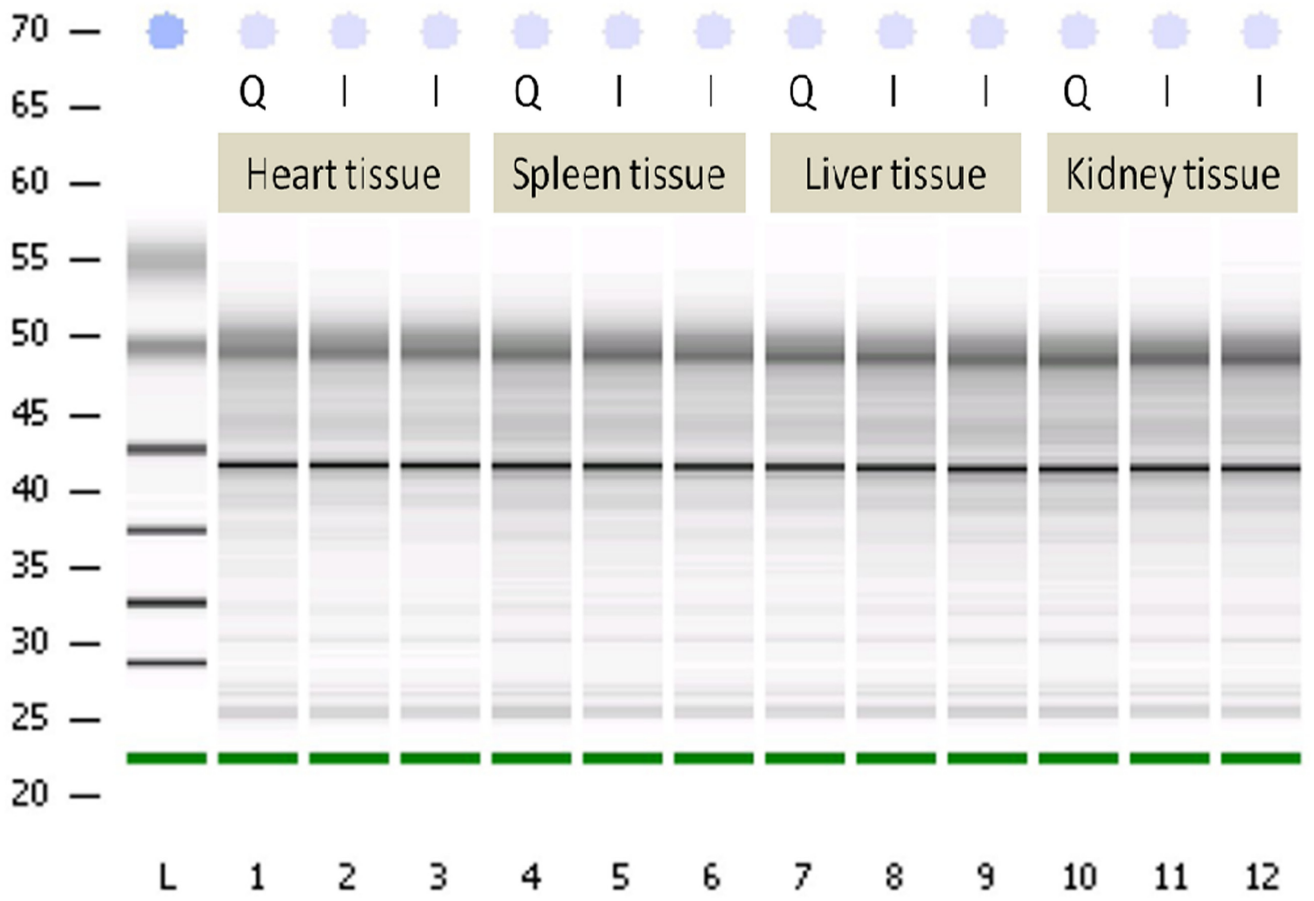
RNA isolation/purification from frozen mouse liver tissue, ITRI (I, duplicate), and Qiagen (Q) samples analyzed by Agilent 2100^®^ gel electrophoresis. Compared with the conventional methods, using single-tube reaction greatly simplified the procedures (see above). Analysis by Agilent 2100^®^ bioanalyzer revealed that the sample qualities were comparable. This demonstrates that by using single-tube reaction the experiment time can be greatly reduced while the nucleic acid quality is maintained.

First we wanted to compare our single-tube nucleic acid purification protocol to those offered by commercialized kits. In single-tube reaction, only one tube was required, which contained sample tissue, lysis buffer and wash buffer. After brief centrifugation two phases were formed: an upper aqueous phase and a lower organic phase containing high-density perfluorohexane. During vortexing, a third immiscible but emulsified phase was generated in which the shearing force was used to break non-covalent bonds between nucleic acids and proteins. Nucleic acids were then adhered to and separated by coated magnetic beads. This replaced the multiple steps of heating, enzyme digestion, washing, nucleic acid binding, and elution in conventional protocols (Figs 1 and 2A). To compare the purity and quality of isolated RNA, the ITRI and Qiagen samples were analyzed by gel electrophoresis (Fig 3). The total RNA yields, the ratio of OD_260_/OD_280_ and OD_260_/OD_230_ (by NanoDrop^®^), and RIN are listed in S3 and S4 Tables. The purity and quality of nucleic acids in ITRI and Qiagen samples were comparable. The mean operating time for ITRI samples was 20 min, whereas that for Qiagen samples was 120 min. Sample duplicates using different RNA collection methods were comparable in RNA yields and integrity (S1 Fig). The ratio of perfluorohexane in the lysis buffer mildly affected final RNA yields and qualities, indicating flexibility (Table 2). The results demonstrate that by using single-tube reaction the experiment time is greatly reduced without sacrificing sample quality.

**Table 2:** The influences of different perfluorohexane ratios on RNA yields and qualities.

Perfluorohexane (w/w%)	Salts (w/w%)	Surfactant (w/w%)	ddH_2_O (w/w%)	Yield (μg/mg)	OD_260_/OD_280_	OD_260_/OD_230_
10	20	1	69	3.5	1.6	1.2
20	20	1	59	3.8	1.75	1.28
30	20	1	49	4	1.8	1.35
40	20	1	39	4.5	1.9	1.5
50	20	1	29	4.6	2	1.6
60	20	1	19	4.2	2.2	1.68
70	20	1	9	3.9	2	1.35

### Single-tube PCR, real-time PCR and multiplex PCR

**Fig 4:**
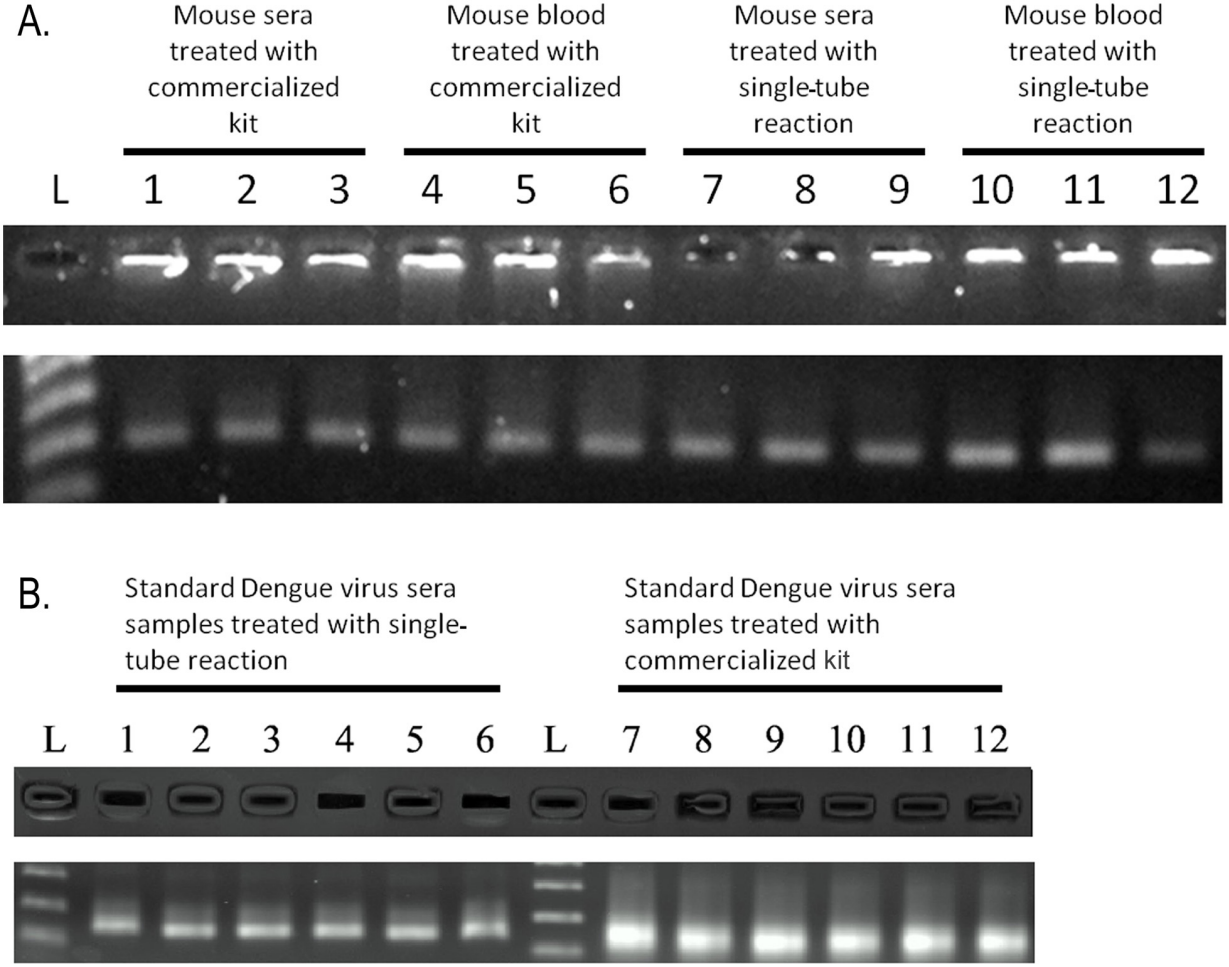
cDNA yields and qualities of single-tube reaction samples. (A) PCR amplification of the *GAPDH* gene, performed in one tube and in one step with the nucleic acid isolation. cDNA yields and qualities of samples amplified using single-tube reactions were comparable to those amplified using commercialized kits (Fermentas Maxima^®^) in two steps after nucleic acid isolation. (B) Standard dengue virus sera samples were tested by RT-PCR to see if trace amounts of specific sequences could be detected. Amplified products from single-tube reaction and commercialized kits were comparable.

**Fig 5:**
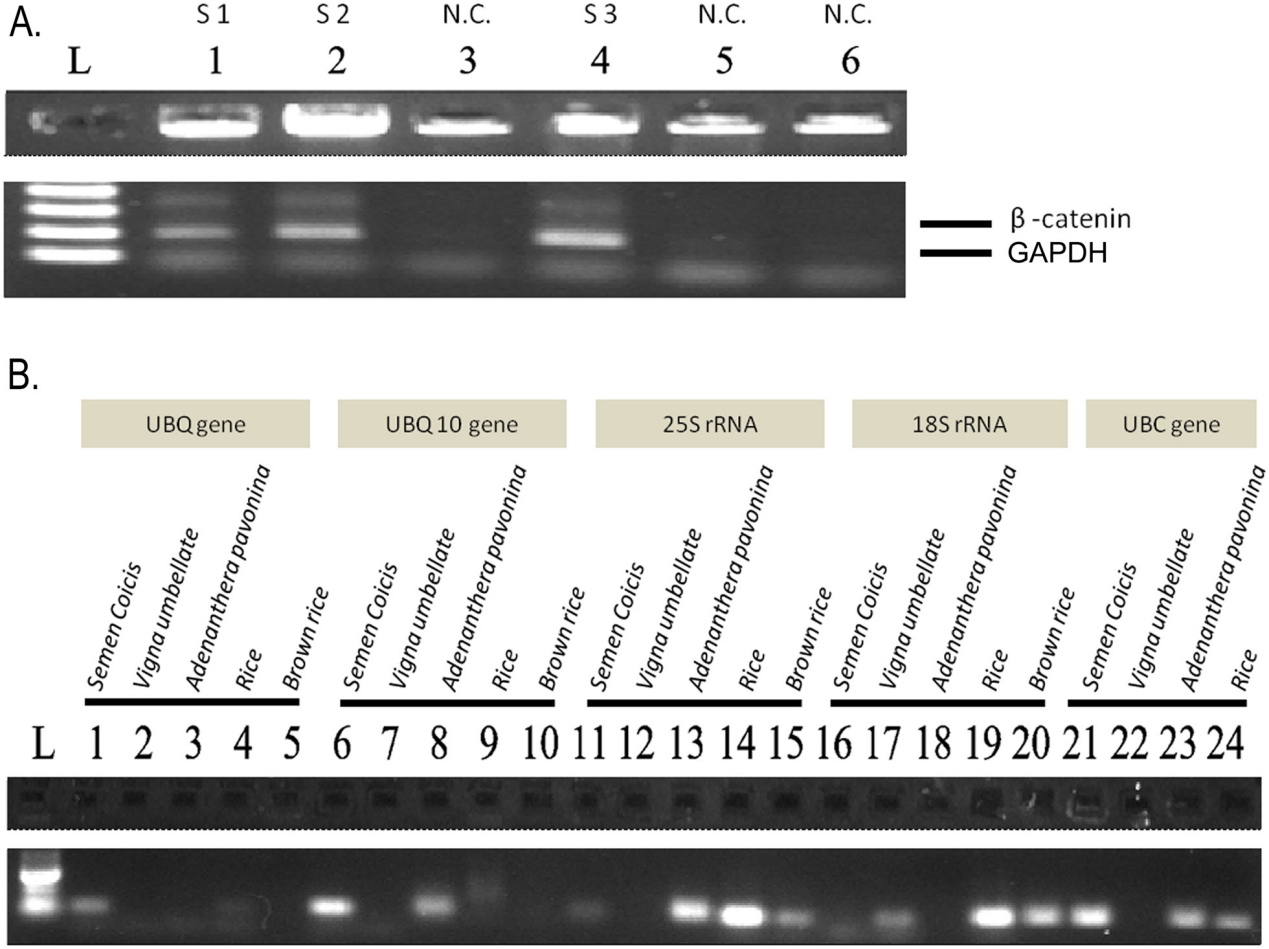
Single-tube multiplex PCR and single-tube PCR detection of selected plant genes. (A) Multiplex PCR reactions were successfully conducted in a single tube (S1.2.3: Sample 1.2.3. N.C.: Negative control). (B) Single-tube amplification for various plant tissues, demonstrating the cell lysis power of PFC in treating plant tissues that contained rigid cell walls.

To prove that PCR could be performed in one tube and in one step, the amplified cDNA products were analyzed. cDNA yields and qualities from single-tube reactions matched those from commercialized kits (Fermentas Maxima^®^) (Fig 4) and could be adequately amplified by real-time PCR (S2 Fig). To further test whether trace amounts of specific sequences could be detected and quantified, standard dengue virus sera were used. Electrophoresis gel run of the amplified solutions of standard dengue virus sera samples prepared using single-tube reaction demonstrated a clear band comparable to those prepared from commercialized kits (Fig 4). Single-tube amplified products from multiplex PCR amplification also demonstrated clear bands on electrophoresis gels (Fig 5), broadening the application of single-tube reaction. To test the cell lysis power of PFC, plant tissues with rigid cell walls were used. Mixtures from various plant tissues were successfully amplified (Fig 5). A wide range of perfluorohexane concentration (30–70%) in mixtures was tolerated, indicating flexibility in PCR reactions.

### Single-tube emulsion PCR

**Fig 6:**
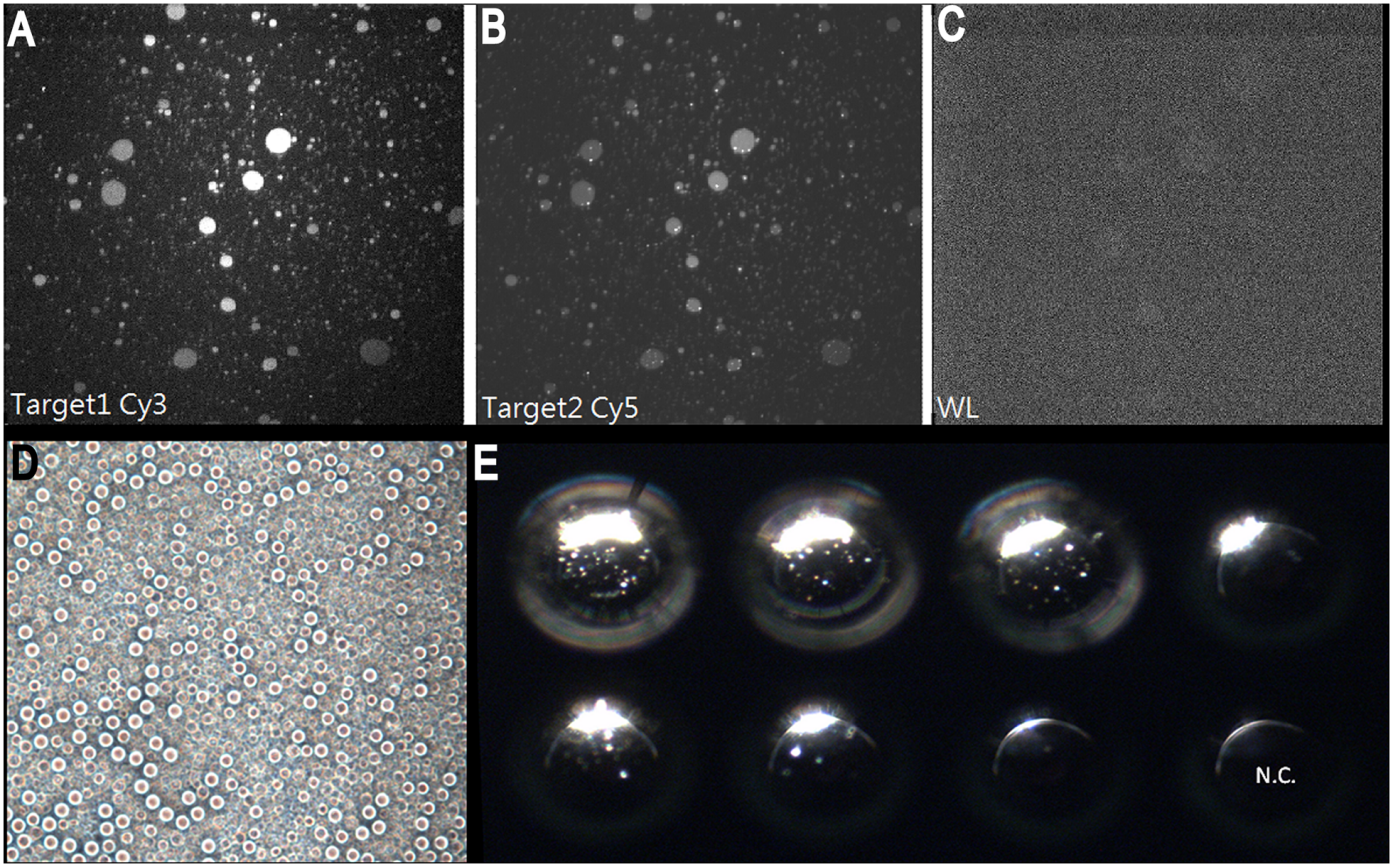
Single-tube emulsion PCR. (A)(B)(C) Target 1 represents the DNA-probe-Cy3 in which the probe 1-Cy3 was labeled. Target 2 represents the DNA-probe-Cy5 in which the probe 2-Cy5 was labeled. WL represents the condition under white light. The results indicate that the biosample treated with a single-tube reaction was in complete oil-ball shapes after being amplified with emulsion PCR, which was beneficial for the next optical analyses. (D)(E) The formation of droplets is shown, which could be placed in arrays. The bright spots inside the droplets are fluorescent emissions from specifically labeled probes. N.C. represents negative control.

To test if our single-tube method could connect with modern next-generation techniques, we conducted emulsion PCR after single-tube nucleic acid isolation (Fig 2C). Emulsion PCR was successfully applied to single-tube reactions. Micrograph and fluorescence emissions are shown in Fig 6.

## Discussion

Developing a robust method for multiple reactions in a single tube is critical for future high-throughput whole-slide in situ techniques. One major advantage of whole-slide in situ methods is that topologic information on diseased tissue can be preserved. Current in situ methods on histopathology slides are either low-throughput, quantitatively limited (e.g., in situ PCR, in situ hybridization, or next-gen immunohistochemistry) [4–7], or high-cost/labor-intensive with a restriction to small areas of sectioned fresh tissue (e.g., in situ mass spectrometry). [8–10] The low reaction capacity on solid-phase tissue also limits ability to perform multiplex reactions with the limited choices of available antibodies. One way to overcome this challenge is to incorporate fluid-phase reactions onto solid-phase tissue sections by gridding it into innumerable non-communicating cells, each of which serves as an independent high-throughput reaction. After treatment, image registry techniques could be used to reconstruct tissue topology.

To perform multiple reaction steps in a single tube, pseudochambers are created so that different steps can be performed in different chambers (phases). Liquid used in organic phase must be high-density, low-polar, immiscible but dispersible in water, chemically stable, and possess low surface tension and low-dielectric constant. By gravity and centrifugation, two different phases (aqueous phase and organic phase) can be formed in a single tube. With intense mixing, a third immiscible but dispersed phase can be generated. Additionally, the low-polar and low-dielectric nature of the compound precipitates nucleic acids, which can be attached to specially-coated magnetic beads and purified by applying an external magnetic field.

To prove this concept, we used perfluorocarbons, hydrocarbons with C-F instead of C-H bonds, which have been used as liquid dielectric or rinsing agents [11], in emulsions, and as nanoparticle oxygen-carrying blood substitutes in medicine. [12] Fluorine is the most electronegative element, resulting in its small size and low atomic polarizability, and the electronegativity difference between carbon and fluorine atoms contributes to the high strength of C-F bonds. In combination, perfluorocarbons generate strong intramolecular forces but very weak intermolecular forces, leading to low surface energies, high hydrolipophobicity, and low dielectric properties. They are good rinsing agents because of their penetrating, insulating, and repellent abilities. Mixed in water, perfluorocarbons form and stabilize emulsions by reducing interfacial tension and the formation of repulsive films on dispersed globules. With intense mixing, shear forces generated between dispersed immiscible globules and water help to break cell membranes and dissociate intracellular molecules, releasing nucleic acids. Perfluorocarbons also have a high binding capacity for proteins but low binding capacity for nucleic acids. [13] In nucleic acid isolation, perfluorocarbons eliminate the interference between proteins and nucleic acids by removing van der Waals forces and blocking non-covalent bond formation. Finally, perfluorocarbons are thermally stable and chemically inert, making them relatively safe agents in thermocycling reactions.

We took advantage of the emulsification, rinsing, and repellent properties of perfluorocarbons for PCR, cell lysis, and nucleic acid isolation. We demonstrated that using perfluorocarbons as a lysis buffer, nucleic acid isolation can be performed in one step and in one tube. Nucleic acids that were isolated were of comparable quality to those isolated using conventional methods. Operating time was reduced, the procedure was simplified, and the method could be effectively applied to various sample types, including intact and ground, liquid and solid, and animal and plant tissues of various sizes. No incubation or enzyme digesting time was needed and the risk of cross-contamination was reduced. Different kinds of perfluorocarbons, including pure or mixed perfluorohexane, perfluorooctane, periodomethane, dichloroperfluorodecane, or commercialized modified/engineered fluids, such as Fluorinert^™^ liquids or Novec^™^ 7500 fluid, for different sample types could potentially be used to optimize reaction performance.

The thermostable and surfactant nature of perfluorocarbon also facilitates the introduction of PCR and real-time PCR into the same tube after nucleic acid isolation. Solution phase in perfluorocarbon micelles are spontaneously formed during PCR heat convection or the oil-surfactant droplets can be prepared separately and added later into the same tube. Our results provide evidence that PCR, multiplex PCR, real-time PCR, and emulsion PCR can be efficiently performed in one tube and one step.

Positively charged, dextran-coated magnetic beads are critical in single-tube reactions. Dextran is water-soluble and high-absorptive, creating another pseudochamber around them in hydrophobic perfluorocarbon-rich fluids. Negatively charged nucleic acids attach firmly to dextran, whereas proteins disaggregate. In contrast, the low-absorptive and non-wetting nature of the beads eliminates other water-soluble substances that do not bind to dextran. Other nanoparticles may also be used, such as ferrite nanoparticles or ultra-small superparamagnetic iron oxide nanoparticles. The magnetic collection device and elution solvents can adapt depending on the characteristics of the magnetic particles and the features of nucleic acids for isolation.

## Conclusions

In conclusion, we have introduced a novel, efficient and robust method, by using perfluorocarbons, to isolate and amplify nucleic acids in one step and in one tube. However, three additional critical steps should be accomplished before a high-throughput in-situ method can be established. These include appropriate image registries to reconstruct tissue topology, an efficient way to distribute carrier oil into hundreds or thousands of independent cells to make droplets, and, finally, an effective way to detect and analyze the signals that are produced (by sequence-specific, color-coded labels). Integrating the four steps may lead to a real digital slide in the future.

## Supporting Information

S1 Fig Sample duplicates using different RNA collecting methods. Sample duplicates using different RNA collecting (purification) methods were comparable in RNA yields and integrity
(TIF)

S2 Fig Real-time PCR in single tube reaction. (A) PCR data of RNA 18S amplification by using ABI standard 18S primers and probes. (B) PCR data of GAPDH amplification by using ABI standard 18S primers and probes. This demonstrated that products of single-tube reaction could be adequately amplified by real-time PCR.
(TIF)

S1 Table Prepare the oil-surfactant mixture. thoroughly mixed in a 50 ml-centrifuge tube at 25°C. (DOCX)

S2 Table Components to form the water phase of emulsion. (DOCX)

S3 Table Total RNA yields, OD_260_/OD_230_ and OD_260_/OD_280_ ratios between ITRI and Qiagen samples using 1mg of mouse liver tissue. (DOCX)

S4 Table RNA integrity number (RIN) measured by Agilent 2100^®^ in different tissue samples. (DOCX)
