# Supplementary figures and images for: Single-Tube Reaction Using Perfluorocarbons: A Prerequisite Step Leading to the Whole-Slide In Situ Technique on Histopathological Slides
Source: PLoS One. 2016 Jun 23;11(6):e0158018. doi: 10.1371/journal.pone.0158018 (PMC4919083; doi:10.1371/journal.pone.0158018)

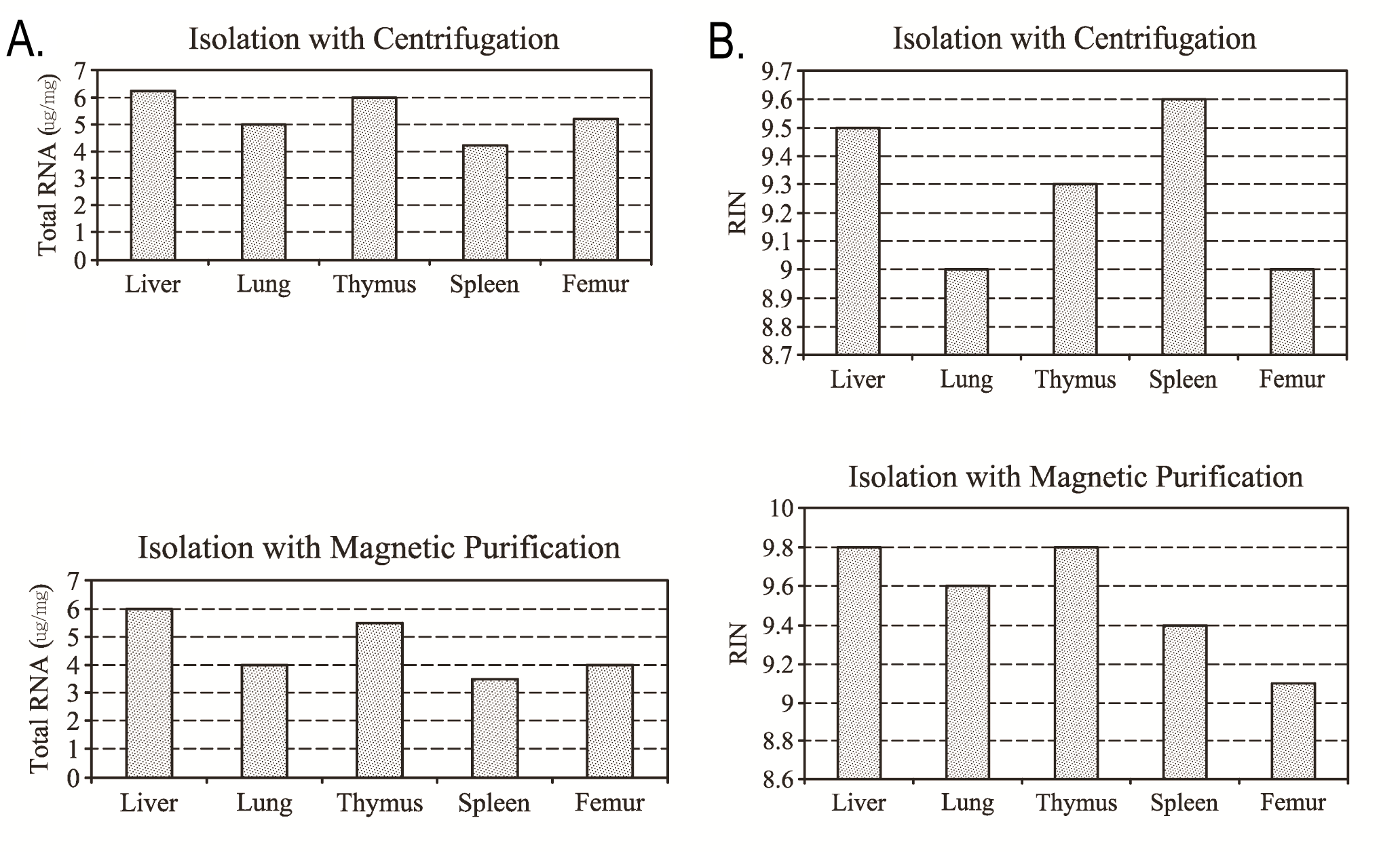

Supplement: S1 Fig — Sample duplicates using different RNA collecting (purification) methods were comparable in RNA yields and integrity (TIF) [file pone.0158018.s001.tif]

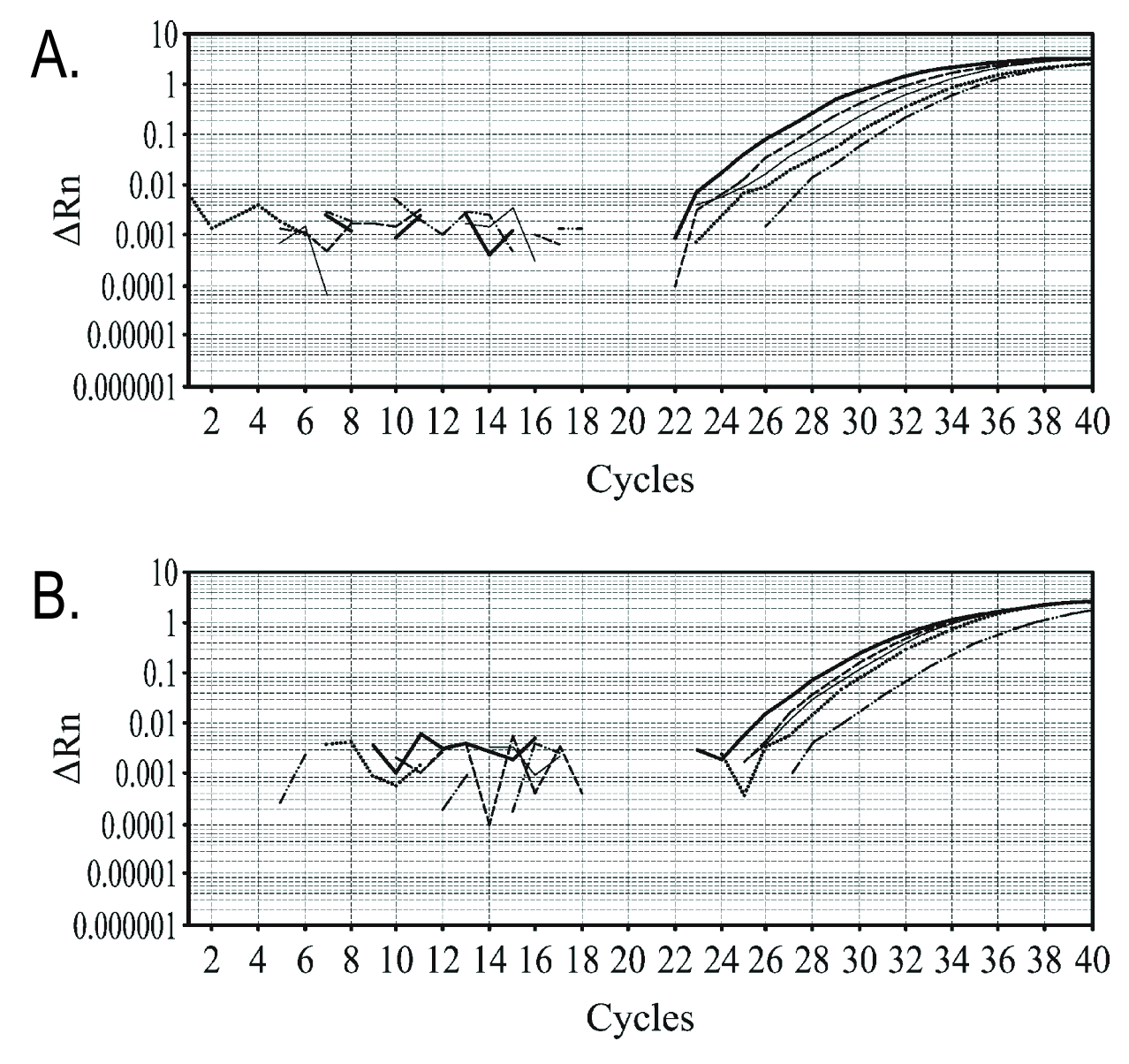

Supplement: S2 Fig — (A) PCR data of RNA 18S amplification by using ABI standard 18S primers and probes. (B) PCR data of GAPDH amplification by using ABI standard 18S primers and probes. This demonstrated that products of single-tube reaction could be adequately amplified by real-time PCR. (TIF) [file pone.0158018.s002.tif]
