# Supplementary material for: Single-Tube Reaction Using Perfluorocarbons: A Prerequisite Step Leading to the Whole-Slide In Situ Technique on Histopathological Slides
Source: PLoS One. 2016 Jun 23;11(6):e0158018. doi: 10.1371/journal.pone.0158018 (PMC4919083; doi:10.1371/journal.pone.0158018)
Supplement: S1 Table — (DOCX) [file pone.0158018.s003.docx]

**S1 Table. Prepare the oil-surfactant mixture. thoroughly mixed in a 50 ml-centrifuge tube at 25° C.**

| **Components** | **Final concentration** |
| --- | --- |
| Span 80 | 4.5% (vol/vol) |
| Tween 80 | 0.4% (vol/vol) |
| Triton X-100 | 0.05% (vol/vol) |
| Fluorescent dye (FAM dye) | 0.01% (vol/vol) |
| Mineral oil | to 1 ml (final volume) |
