# Supplementary material for: Single-Tube Reaction Using Perfluorocarbons: A Prerequisite Step Leading to the Whole-Slide In Situ Technique on Histopathological Slides
Source: PLoS One. 2016 Jun 23;11(6):e0158018. doi: 10.1371/journal.pone.0158018 (PMC4919083; doi:10.1371/journal.pone.0158018)
Supplement: S2 Table — (DOCX) [file pone.0158018.s004.docx]

**S2 Table. Components to form the water phase of emulsion.**

| **Components** | **Volume** |
| --- | --- |
| 10× Clone Pfu buffers | 1 μl |
| BSA (100 g/l) | 1 μl |
| Forward primers (10 μM) | 1 μl |
| Reverse primers (10 μM) | 1 μl |
| dNTPs (5 mM) | 2 μl |
| Pfu Turbo DNA polymerase | 1 μl |
| Probe 1-cy3 (100 nm)^a^ | 0.5 μl |
| Probe 2-cy5 (100 nm)^a^ | 0.5 μl |
| Template DNA^b^ | ≦10^9^ molecules (1.66 fmol) |
| Water | to 10 μl (final volume) |

^a^ These allele-specific probes could be designed to each contains a base complementary to the SNP or mutated nucleotide in the target (interested) gene. An addition biotin-labeled capture sequence could be designed to hybridize to a sequence in the target gene that does not contain a SNP or mutation of interest. The biotin-labeled capture could be conjugated to streptavidin-coated beads for isolation. An example for detection of *VKORC1* gene: 5′-biotin-c12-CTTTGGAGACCAGCCCATGGGGACAGAGTCAGA (biotin-labeled capture)

5′-Cy3-CACATTTGGTCCATTGTCATGTGT (Cy3-labeled SNP probe, *VKORC1* rs 7294)

5′-Cy5-ACATTTGGTCCATTGTCATGTGC (Cy5-labeled SNP probe, *VKORC1* rs 7294)

^b^ Control oligonucleotides that could be labeled by Texas Red or FAM dyes.
