# Supplementary material for: Single-Tube Reaction Using Perfluorocarbons: A Prerequisite Step Leading to the Whole-Slide In Situ Technique on Histopathological Slides
Source: PLoS One. 2016 Jun 23;11(6):e0158018. doi: 10.1371/journal.pone.0158018 (PMC4919083; doi:10.1371/journal.pone.0158018)
Supplement: S3 Table — (DOCX) [file pone.0158018.s005.docx]

**S3 Table. Total RNA yields, OD_260_/OD_230_ and OD_260_/OD_280_ ratios between ITRI and Qiagen samples using 1mg of mouse liver tissue.**

|  | Total RNA yield (μg/mg) | OD_260_/OD_280_ | OD_260_/OD_230_ |
| --- | --- | --- | --- |
| ITRI Sample | 6.04 | 2.08 | 2.18 |
| Qiagen Sample | 5.99 | 1.95 | 2.16 |
