# Supplementary material for: Single-Tube Reaction Using Perfluorocarbons: A Prerequisite Step Leading to the Whole-Slide In Situ Technique on Histopathological Slides
Source: PLoS One. 2016 Jun 23;11(6):e0158018. doi: 10.1371/journal.pone.0158018 (PMC4919083; doi:10.1371/journal.pone.0158018)
Supplement: S4 Table — (DOCX) [file pone.0158018.s006.docx]

**S4 Table. RNA integrity number (RIN) measured by Agilent 2100® in different tissue samples.**

|  | Tissue types | | | | | | | | | | | |
| --- | --- | --- | --- | --- | --- | --- | --- | --- | --- | --- | --- | --- |
|  | Heart | | | spleen | | | liver | | | kidney | | |
| Lane No. | 1 | 2 | 3 | 4 | 5 | 6 | 7 | 8 | 9 | 10 | 11 | 12 |
| RIN | 6.0 | 7.8 | 8.0 | 7.3 | 7.7 | 7.8 | 7.8 | 7.5 | 7.5 | 7.5 | 7.6 | 7.7 |
